# Editorial: Shared responses and individual differences in the human brain during naturalistic stimulations

**DOI:** 10.3389/fnhum.2023.1201728

**Published:** 2023-05-05

**Authors:** Zhishan Hu, Xin Di, Zhi Yang

**Affiliations:** ^1^Neuroimaging Core, Shanghai Mental Health Center, Shanghai Jiao Tong University School of Medicine, Shanghai, China; ^2^Department of Biomedical Engineering, New Jersey Institute of Technology, Newark, NJ, United States; ^3^Beijing Key Laboratory of Mental Disorders, National Clinical Research Center for Mental Disorders and National Center for Mental Disorders, Beijing Anding Hospital, Capital Medical University, Beijing, China; ^4^Advanced Innovation Center for Human Brain Protection, Capital Medical University, Beijing, China

**Keywords:** shared response, individual difference, naturalistic stimulation, psychiatry, education

Conventional functional brain imaging experiments present stimuli in a controlled and repetitive manner based on predefined protocols. Although offering crucial insights into brain function, their real-world relevance is uncertain. Investigating brain activity in naturalistic settings can help fill the gaps (Nastase et al., 2019; Finn et al., 2020).

Following Hasson et al.'s pioneering work on shared brain activity during naturalistic movie viewing (Hasson et al., 2004), researchers have made substantial progress in this field. On one hand, researchers have found that shared brain activity can reflect the multifaceted cognitive and emotional information processing properties of the human brain, such as shared understanding, empathy, mathematical ability (Cantlon and Li, 2013), cognitive development (Di and Biswal, 2022), education (Hu et al., 2019; Meshulam et al., 2021), interpersonal relationships (Parkinson et al., 2018), and even brain characteristics of mental illness (Hasson et al., 2009; Zhang et al., 2022; Jin et al., 2023). On the other hand, shared brain activity has emerged as a valuable tool for identifying individual differences (Finn et al., 2020; Di et al., 2023). By detecting the spatiotemporal characteristics of the emergence of shared brain activity in healthy groups or reference groups, researchers can construct reference or normative models that enable them to make inferences about the degree of deviation from the characteristics of brain activity in individuals (Yang et al., 2020). Naturalistic stimuli with rich social-emotional information are able to express individual differences stably in many brain regions (Gao et al., 2020), providing important support for individualized inferences based on brain responses.

The current Research Topic encompasses studies that employ a naturalistic paradigm and a variety of brain imaging and behavioral measuring devices, including functional near-infrared spectroscopy (fNIRS), electroencephalograph (EEG), surface electromyography (sEMG), virtual reality device, and eye-tracking glasses. By leveraging the signals from these devices and utilizing cutting edge data analysis approaches, their studies have significantly deepened our understanding of how the brain responds to stimuli in real-world settings.

This collection includes two review articles. Jääskeläinen et al. provided an overview of inner experiences sampling techniques and the joint analyses of these measurements with neuroimaging data. Their review highlighted the potential for developing neurophenomenological frameworks to understand the relationship between inner experiences and their neural underpinnings. Liu et al.'s review focused on the parent-child inter-brain neural synchrony (INS) and its impact on children's social development. Their review demonstrated stronger parent-child INS as compared to the stranger-child dyads, discussed situational factors that influence the parent-child INS, and explored how INS relates to behavioral tendencies and individual features of both parents and children.

The studies featured in this collection showed that stimuli with more naturalistic properties can effectively elicit shared response. Using the sound from the natural environment, for example, Wang Y. et al. found positive emotional naturalistic sounds could modulate visual spatial attention. The event-related potential (ERP) revealed enlarged N1 amplitudes after positive sounds than neutral sounds. Similarly, in a naturalistic setting, Mueller et al. used sEMG to measure the facial muscles during mimic movements, and found that facial movement tasks evoked shared response in the complex network of facial muscles rather than activating a single muscle. Further, Boustani et al. employed virtual reality technique to study the brain-computer interface (BCI) performance in a real-world environment. When the additional auditory cues were presented, they observed increased classification accuracy for a detection task using brain activity features, compared to the visual-only condition, highlighting the importance of multisensory integration in BCI performance.

The naturalistic paradigm can be valuable in educational settings. For instance, Zhang et al. used a prisoner's dilemma game combined with the fNIRS-based hyper-scanning technique to explore the neural basis underlying team collaborative decision-making. Higher INS was identified in the right inferior frontal gyrus during team collaborative decision-making. Similarly, Soares et al. used fNIRS and eye-tracking to examine the physiological responses of children during a mental rotation test in a naturalistic education environment. They observed significant activation on the dorsolateral prefrontal cortex and increased pupil diameter during the task. The brain and physiological indicators discovered in these studies can be valuable for neurocomputational modeling of cognition in a naturalistic environment. In addition, Pereira Soares et al. found that individual differences in real life bilingual language experience affect oscillatory dynamics of inhibition and cognitive control.

The investigation of shared response and individual differences in the human brain in naturalistic environments holds potential for research in psychiatry. Wang K. et al. analyzed EEG signals in patients with major depression disorder (MDD) and healthy controls (HC) while freely listening to music. They found that both groups triggered similar brain dynamics when listening to music, but MDD patients also exhibited some alterations in brain oscillatory network characteristics during music perception. These findings could potentially aid in the clinical diagnosis and treatment of MDD patients.

In conclusion, the utilization of naturalistic paradigms using diverse brain imaging and behavioral measuring devices can provide valuable insights into shared responses and individual differences in the human brain. The research articles in this collection highlight the potential of neurophenomenological frameworks, the significance of multisensory integration in brain-computer interface performance, and the applicability of naturalistic paradigms in educational and psychiatric settings.

## Author contributions

All authors listed have made a substantial, direct, and intellectual contribution to the work and approved it for publication.
